# The use of colposcopy for triage in HPV-positive women aged 65 years and older

**DOI:** 10.1007/s00404-023-07281-5

**Published:** 2023-12-11

**Authors:** E. Kuenkel, A. Jaeger, I. Bohlmann, F. Bergauer, C. Kuehler-Obbarius, K. Prieske, K. Maass-Poppenhusen, B. Schmalfeldt, L. Woelber

**Affiliations:** 1 Dysplasia Unit Women’s Practice Heussweg, Frauenarztpraxis und Dysplasie-Einheit Heussweg, Heussweg 37, 20255 Hamburg, Germany; 2 Department of Gynecology, University Medical Center Hamburg–Eppendorf, Martinistr. 52, 20246 Hamburg, Germany; 3 Dysplasia Unit at Jerusalem Hospital, Moorkamp 2-6, 20357 Hamburg, Germany; 4 Dysplasia Munich, Nymphenburger Str. 77, 80636 Munich, Germany; 5 Department of Gynecology, University Hospital Campus Kiel, Arnold Heller Str. 3, 24105 Kiel, Germany; 6 Cytologylaboratory Dr. Med. Kühler-Obbarius, Fangdieckstr. 75a, 22547 Hamburg, Germany

**Keywords:** NILM, HPV persistence, Triage, Elderly

## Abstract

**Purpose:**

Persistent high-risk HPV infection is associated with an elevated risk for prevalent CIN II + despite normal cytology (NILM). Our study aims to evaluate the clinical relevance of a persistent high-risk HPV infection without cytologic changes in women aged ≥ 65 and to determine the role of colposcopy for triage in these cases.

**Methods:**

211 patients aged ≥ 65 with persistent HPV infection and normal cytology (NILM) who presented for colposcopy at five certified centers between January 2021 and April 2022 were included in the study. Colposcopic findings, HPV subtypes, when available, histology and p16/Ki67 staining were assessed as well as individual risk factors such as smoking and previous HPV-related surgery.

**Results:**

87.7% (185/211) of the included women had a type 3 transformation zone. In 83.4% (176/211), a biopsy was taken [thereof 163 endocervical curettages (ECC)]. In 35/211 women (16.6%), sampling was not possible during colposcopy due to an inaccessible cervix, pain during examination or obliteration of the cervical canal. Out of these, 6 women received a diagnostic excision. CIN II + was detected in 10.6% of all histologies (excisional or biopsy) (20/182). 50% of the women with a CIN II + where HPV 16 positive. Taking only the women diagnosed with CIN III or AIS into account, (*n = *12) 75% were HPV 16 positive. Interestingly, 80% of the women with CIN II + had an abnormal cytology when repeatedly taken during colposcopy, vice versa an endocervical lesion was diagnosed in 53% of women with abnormal repeat cytology (27/51).

**Conclusion:**

The prevalence of CIN II + in women is ≥ 65 with persistent hr HPV infection but NILM cytology is similar to that in younger women. However, more than 85% of the women have a type 3 transformation zone. Colposcopy is, therefore, not helpful to diagnose the women who need treatment in this age group.

## What does this study add to the clinical work?

**Table:** 

HPV-positive women with NILM cytology may have a severe dysplasia that is much more difficult to diagnose. As colposcopy does often not reveal the changes, additional diagnostic methods should be applied and further evaluated.	

## Introduction

In 2020, cervical cancer screening in Germany was adapted from a solely cytology-based screening to a co-testing approach. All women aged ≥ 35 years are now offered a testing for hr (high risk) HPV every 3 years combined with cytology (co-testing). When a hr HPV infection is detected, co-testing is repeated after 1 year. The new screening method results in a large group of HPV-positive women with NILM cytology [1]. In case of hr HPV persistence, women are referred to colposcopy for further assessment. The immediate risk of CIN II + in this specific group ranges from 4 to 15% in women aged 35–65 years dependent on the HPV type detected [2–4]. Nevertheless, data on the incidence and impact of hr HPV in elderly women are scarce. A Danish study including 108.585 women showed, that only 4.1% of all women aged 65 years and older were HPV positive, compared to 6–8% in women aged 35–65 [5]. Currently, there are limited data on the significance of a persistent hr HPV infection in women of older age and their consecutive residual live time risk of cervical cancer [6, 7]. It is well known that cervical carcinoma incidence has two peaks, one between 35 and 40 years and one between 65 and 80 years [5]. Of note, cervical cancer of older women is predominantly detected at advanced stages [8, 9].

Anatomical changes after menopause make the currently available diagnostic approaches more challenging. the transformation zone (TZ) of postmenopausal women is often located in the upper part of the cervical canal and cannot be seen [10]. Colposcopy as well as the sampling of the transformation zone with biopsy is thus more difficult. Targeted biopsy is impossible and endocervical curettage has a poor sensitivity [11, 12]. It is also well known that cytology alone has a reduced sensitivity in postmenopausal women [7, 13, 14].

Aim of the current study is, therefore, to assess the prevalence of a hr HPV infection in elderly women ≥ 65, who are taking part in the organized German screening program. The clinical relevance of a persistent hr HPV infection and the diagnostic measures specified in the screening algorithm should be evaluated for the subgroup with normal pap smear (NILM).

## Methods

This is a retrospective multicenter analysis. We analyzed all patients ≥ 65 years that presented with a repeatedly positive hr HPV screening test (for ≥ 1 year) and normal cytology between January 2021 and April 2022 at five certified dysplasia centers in Germany (University Medical Center Hamburg-Eppendorf, University Hospital Campus Kiel, Schleswig Holstein, Dysplasia-Unit Heussweg, Hamburg, the Dysplasia Unit at the Jerusalem Hospital in Hamburg and in the Dysplasia-Unit Dyplasie München in Munich). All women included were taking part in the organized cervical screening program. Women who had a hysterectomy were excluded from the study. The collected data are based on quality assurance data from the participating centers. All women underwent a colposcopy. For diagnostic colposcopy, IFCPC nomenclature was applied [15]. Cytology results were initially classified using München III classification. The results were then transferred into the international Bethesda 2014 classification. Immunologic staining (p16/Ki67) was performed with CINtec plus, Roche Diagnostics, if indicated by the colposcopist. Cervical curettage and/or targeted biopsies were taken when indicated by the examiner. The transformation zone was assessed, and the colposcopic quality and the quality of the histologic material were also evaluated. We assessed, whether the biopsies contained squamous epithelium and columnar epithelium (sufficient) or whether they contained only squamous epithelium (non-sufficient). The biopsy was classified as non representative when there were very little cells and predominantly mucus. When more than one biopsy was obtained, the most severe result was used for analyses. Pathological evaluation was done by specialized gyne-pathologists from the participating centers. We also assessed data about parity, previous operations, immunosuppression and smoking. In addition, data regarding the prevalence of hr HPV infection in women ≥ 65 since the start of the screening in January 2020 were retrieved from one of the participating centers with adjacent cytology laboratory, the Reference Center for Cytology in Hamburg (20,500 cytologies per year). HPV tests were performed with COBAS testing from Roche Diagnostics.

## Results

In 2020, 25,131 co-tests in women aged ≥ 35 were performed at the Reference Center for Cytology Hamburg with the start of the adjusted screening program. 4327 women were 65 years and older (17.2%). 1084 women out of the whole group were HPV positive (4.4%). Only 119 of the women ≥ 65 were HPV positive (2.8%).

**Fig. 1:**
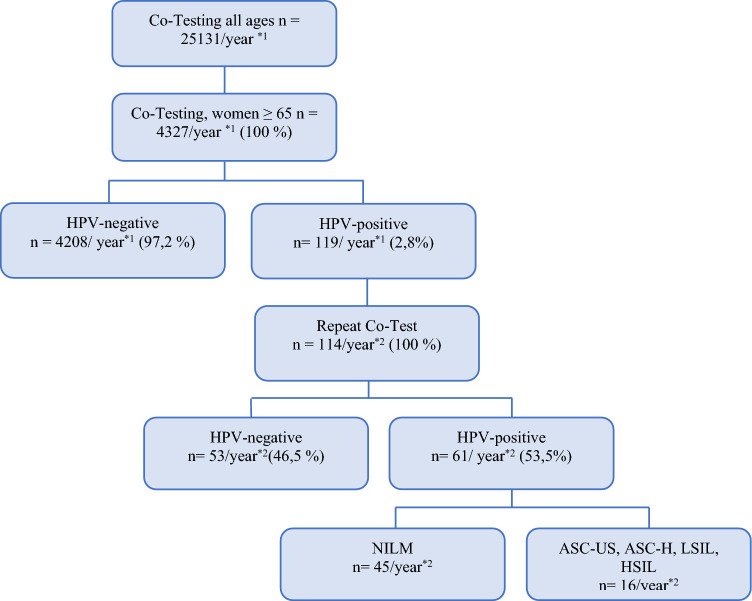
Flow chart showing number of HPV-positive elderly women

In 2021, 1127 repeat co-tests in previously hr HPV positive women aged ≥ 35 were performed. 114 were taken from the women ≥ 65 (10.1%). 61 of the repeat co-tests were persistently HPV positive (53.5%). For numbers of HPV-positive women, see Fig. 1.

At the Dysplasia-Unit Heussweg, 1277 diagnostic colposcopies due to persisting HPV infection or abnormal cytology were performed. Out of those, only 45 (3.5%) women were ≥ 65 years and had a normal cytology. To expand this specific subgroup, we included data from 4 centers. We analyzed 211 women aged 65 years and older with normal cytology (NILM) and persistent HPV high-risk infection who had attended colposcopy after the abnormal screening result. None of the women had had an HPV-vaccination.

**Fig. 2:**
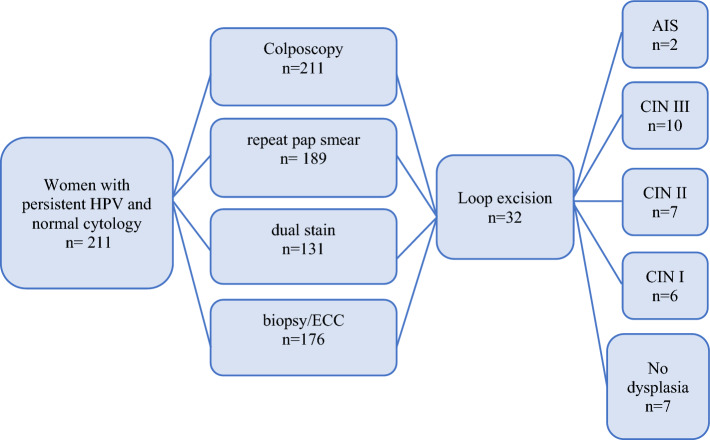
Diagnostic measures performed in the study cohort (*n = *211)

For patients’ characteristics, distribution of transformation zone types and colposcopic findings as well has distribution of HPV types, see Table 1 and Fig. 2.

**Table 1:** Patients’ characteristics

	211 (100%)
Median age	71
Previous surgery HPV related (e.g., LLETZ, VIN/VAIN/AIN)	20 (9.5%)
Smoking	24 (11.4%)
Transformation zone	
Type 1	2 (0.9%)
Type 2	24 (11.4%)
Type 3	185 (87.7%)
Colposcopy	
Not adequate	16 (7.6%)
Normal	157 (74.4%)
Minor change	31 (14.7%)
Major change	7 (3.3%)
HPV types	
Type 16	50 (23.7%)
Type 18	20 (9.5%)
Other types	157 (74.4%)
Repeat cytology	
NILM	138 (65.4%)
ASC-US	10 (4.7%)
ASC-H	16 (7.6%)
ACG-FN	5 (2.4%)
LSIL	8 (3.8%)
HSIL	12 (5.7%)
Not taken	22 (10.4%)
Histology	
Benign	144 (68.3%)
CIN I	18 (8.5%)
CIN II	8 (3.8%)
CIN III	10 (4.7%)
AIS	2 (1.0%)
Not taken	29 (13.8%)
Dual stain	
Positive	25 (11.8%)
Negative	106 (50.3%)
Not taken	80 (37.9%)

**Fig. 3:**
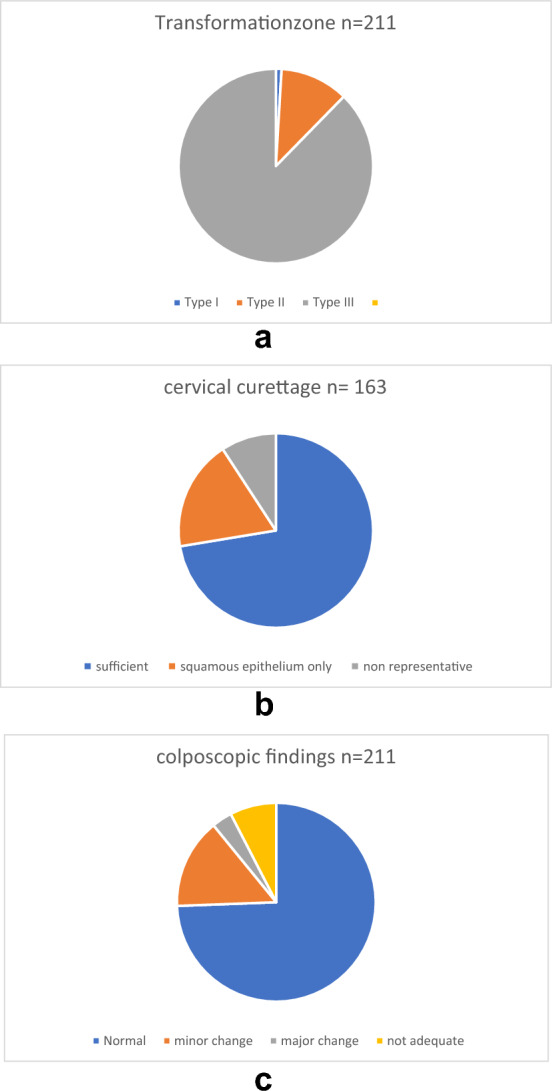
**a** Transformation zone. **b** Cervical curettage. **c** Colposcopic findings

In 16 women (7.6%), colposcopy was unsatisfactory as the cervix could not be assessed properly due to scarring, atrophy or other anatomic changes. For colposcopic findings, see Fig. 3a–c.

Under colposcopic view, 189 (89.6%) women had a new cytological smear taken. Out of all repeat smears with abnormal result (*n = *51), 27 women had an abnormal histology CIN I–CIN III (53% 27/51). Immunohistochemical staining of cytology with dual staining was performed in 131 women out of all 211 (62%). Of the 131 p16/Ki67 stainings, 25 were positive (11.8%) and 106 negative (50.3%). Relation to histological results is presented in Table 2.

**Table 2:** Diagnostic findings in pts with HPV-associated lesions

Histology	HSIL *n = *20	LSIL *n = *18
Colposcopy normal	11	11
Minor change	4	6
Major change	5	1
Cytology < ASC-US	2	3
Cytology > ASC-US	16	10
Histology at colposcopy abnormal	16	15
Dual stain pos	10	8
Dual stain neg	5	1
HPV 16	9	5
HPV 18	1	2
HPV-ot	11	11
Smoking	5	0
Surgery HPV rel	1	1

Histology was assessed in 182 (86.3%) women. 176 women (85.3%) had a sample taken during the diagnostic colposcopy. In 35 women, no biopsy was taken during colposcopy, because the examination was unsatisfactory. Out of these 35 women, 6 received a diagnostic conization. Altogether, 29/211 women remained without histological clarification (13.8%).

145 women received a cervical curettage only, 18 women received curettage and targeted cervical biopsies, and 13 women received only cervical biopsy during the diagnostic colposcopy. 47 of the cervical curettages contained only squamous epithelium, glandular cells were not present or there was only mucus. They were thus not representative. The histological results of the cervical biopsies were distributed as follows: 17 CIN I, 6 CIN II, 7 CIN III, 2 AIS, and 144 no dysplastic lesions. Among the benign findings, there were 16 metaplasias, 15 cervicitis, and 4 condylomatous lesions.

No cervical carcinoma was diagnosed. Table 3 shows all patients undergoing a conization (diagnostic or therapeutic; *n = *32) and their corresponding results.

**Table 3:** Women undergoing conization and corresponding results

Results conization	CIN I	CIN II	CIN III	AIS	benign
*n = *32	*n = *6	*n = *7	*n = *10	*n = *2	*n = *7
Median age	72	70	68	70	69
Major change	0	1	3	1	0
Minor change	1	1	3	0	0
Normal/not adequate	5	5	4	1	7
HPV 16	0	0	7	2	2
HPV 18	2	1	0	0	0
HPV-ot	4	6	3	0	5
Cytology < ASC-US	0	0	2	0	3
Cytology > ASC-US	6	7	7	2	4
Dual stain pos/neg/n.a	0/ 4 /2	4 /3	4 / 2 /4	1 /0 /1	4/ 3 /0

## Discussion

Data on HPV prevalence and risk of dysplasia in older women are rare. Swedish and Danish data show a low incidence with around 3% of persistent HPV infection in elderly women [16, 17]. In one study with limited patient numbers elderly women with persistent positive hr HPV infection were described to have an increased risk of developing high-grade dysplasia [17]. A recent Swedish study offering a self-sampling HPV Test to women aged 65 and older also showed a very low rate of persistent hr HPV infections with only 1.3%. In the same study, it was also shown that women with a newly acquired hr HPV infection had a higher risk for LSIL, compared to women with a persistent HPV infection of longer duration [18].

In our cohort, we examined women ≥ 65 years, who remained HPV positive at the repeat test taken a year later. The overall prevalence of persistent HPV infection was low (2.6%) and therewith comparable to the smaller cohorts. We found a slightly higher clearing rate of 46.5% for HPV infection than in the Hermansson’s study, which also looked at patients with a repeat positive HPV test performed after an average 3.5 months and showed a clearing rate of 37.2% most likely due to the narrower time interval between the two tests [16]. In our cohort, it is unknown at what point the HPV infection was acquired. Since the HPV-based screening started in 2020, some women may have had a persistent HPV infection for years.

Although in our cohort the prevalence of a persistent HPV infection was low, the prevalence of CIN II + , when persistent hr HPV infection was diagnosed, was similar to that described in younger women (10%) [19, 20].

Cytological screening in postmenopausal women is known to be problematic [7, 13, 14]. The sensitivity of the primary cytological screening is low with 55% [4]. However, 80% of the included women with CIN II + had an abnormality when the cytology was repeated at the centers under visual inspection, which than led to the decision to perform an excision. Almost 90% of the women examined by an experienced colposcopist had a non-visible type 3 transformation zone. Colposcopy is, therefore, of limited value in this age group, since the dysplastic lesion is often located in a non-visible area. It might help to take a targeted cytological smear or an ECC under colposcopic vision. Of note, the histological samples taken during colposcopy could not be adequately assessed in 47 cases (22.3%). In view of these obstacles, the question arises whether diagnostic excision should be offered to elderly women with HPV persistence in the absence of adequate histological clarification and type 3 transformation zone. A recent study showed that a persistent hr HPV infection with a median time of HPV persistence of 20 months might lead to a HSIL in 32% [21]. Therapy by means of excision could be useful to avoid lengthy controls with limited tools. The importance of the cervix for pregnancy is no longer relevant in this age group. Instead, increased diagnostic certainty would be achieved and repeat painful examinations avoided. A Danish study recommends considering diagnostic conization in older women with a non-visible transformation zone or insufficient histology in case of persisting HPV infection [21–23]. In our study, almost 10% of women with persistent HPV infection had a CIN II + and were thus at risk of developing cervical cancer. Diagnosis was difficult and not always possible with colposcopy and biopsy. One has to keep in mind that after surgery scaring, stenosis and shortening of the cervix will further impede regular pap smears and might even trigger secondary hysterectomy [24]. Our results show that diagnostic methods as colposcopy, histology and cytology, any of these procedures are less accurate in elderly women. The location of the transformation zone makes it often impossible to clarify by colposcopy whether a HSIL is present or not. The role of endocervicoscopy needs to be evaluated. In our cohort of elderly women, the procedure is difficult to perform due to the very narrow cervical os or cervical stenosis. Current literature reveals that this method has only been applied to women of younger age with TZ3 [25, 26]. A weakness of our study is the retrospective nature. Additional research is needed to stratify the risks of these women further. Presentation of these women in certified centers is recommended as the diagnostics needs an experienced specialist. Most European countries have established an HPV-based screening with an upper age limit because of the harm caused by false-positive results. Overtreatment of abnormal results could exceed the benefits of the screening in elderly women [27]. In Germany, there is no upper age limit for cervical cancer screening, but it is discussed whether it should be established and at which age. Today, the evidence for effectiveness of screening beyond the age of 65 years is limited, based on solely observational studies. There is a controversy about the significance of a negative exit test [27, 28]. Criteria to discuss an age of cessation include previous screening history, expected life span and general health [28].

## Conclusion

The prevalence of hr HPV infection in elderly women is low. Nevertheless, their risk of a HSIL is similar to that of younger women. Colposcopy is less effective in these women. Diagnostic methods under consideration of the special anatomic features in this age group must be developed further.

## Data Availability

The data that support the findings of this study are available from the corresponding author, EK, upon reasonable request.
